# Impact of Plant Origin on Eurasian Propolis on Phenolic Profile and Classical Antioxidant Activity

**DOI:** 10.3390/biom11010068

**Published:** 2021-01-06

**Authors:** Piotr Okińczyc, Jarosław Widelski, Jakub Szperlik, Magdalena Żuk, Tomasz Mroczek, Krystyna Skalicka-Woźniak, Zuriyadda Sakipova, Gabriela Widelska, Piotr Marek Kuś

**Affiliations:** 1 Department of Pharmacognosy and Herbal Medicines, Wroclaw Medical University, 50-556 Wrocław, Poland; piotr.kus@umed.wroc.pl; 2 Department of Pharmacognosy with Medicinal Plant Unit, Medical University of Lublin, 20-093 Lublin, Poland; jaroslaw.widelski@umlub.pl; 3 Faculty of Biotechnology, University of Wroclaw, 50-383 Wrocław, Poland; magdalena.zuk@uwr.edu.pl; 4 Independent Laboratory of Natural Products Chemistry, Medical University of Lublin, 20-093 Lublin, Poland; tomasz.mroczek@umlub.pl (T.M.); kskalicka@pharmacognosy.org (K.S.-W.); 5 School of Pharmacy, S.D. Asfendiyarov Kazakh National Medical University, Almaty 050000, Kazakhstan; sakipova.z@kaznmu.kz; 6 Department of Inorganic Chemistry, Medical University of Lublin, 20-093 Lublin, Poland; gabrielachodun@yahoo.com

**Keywords:** propolis, Eurasia, antioxidant, DPPH, FRAP, ORAC, HPLC, UPLC, DAD, MS, chemometry

## Abstract

Propolis is a bee product with known medical properties, including antioxidant activity. The scope of the study is profiling 19 different Eurasian propolis samples (mostly from Russia and Kazakhstan, Kyrgyzstan, Poland, Ukraine, and Slovakia). Profiles of propolises were investigated by ultra-high-performance liquid chromatography–diode array detector–mass spectrometry (UPLC-DAD-MS). Classical antioxidant properties, which are based on electron donation mechanism, were assessed by DPPH, ferric reducing antioxidant power (FRAP), and oxygen radical absorbance capacity (ORAC) assays. Total phenolic and flavonoid contents were also evaluated by colorimetric tests. Most of the samples exhibited significant content of polyphenols (from 30.28 to 145.24 mg GAE/g of propolis) and flavonoids (from 10.45 to 82.71 mg GAE/g of propolis). Most of the propolis samples exhibited potent antiradical (DPPH test—from 8.83 to 64.47 mg GAE/g of propolis) and reducing activity (FRAP test—from 0.08 to 1.17 mmol Fe^2+^/g of propolis). Based on the occurrence of marker compounds, propolis samples were classified as poplar, aspen–birch, aspen–poplar, and aspen–birch–poplar type. Main markers present in propolis of poplar (e.g., chrysin, pinocembrin, galangin, and 3-*O*-acetyl-pinobanksin), birch (ermanin and acacetin) and aspen (2-acetyl-1,3-di-*p*-coumaroylglycerol) origin were used. DPPH, FRAP, and ORAC tests results were correlated with flavonoids, total polyphenols, or the polyphenols other than flavonoids content. In term of activity, poplar propolis type was variable, while aspen–birch–poplar type usually exhibited high DPPH and FRAP activity.

## 1. Introduction

Propolis (bee glue) is a bee product known to possess multiple medicinal properties such as antimicrobial, anti-inflammatory, antioxidant, cytotoxic, and wound-healing [1]. Today, these properties are still used in traditional and experimental medicine.

The main sources of biologically active substances in propolis are plant exudates [1,2]. 

Due to high variability of flora around the world and other factors, chemical composition of propolis as a substance is unstable [2]. However, bees demonstrate notable preferences in relation to collected plant exudates [3]. Therefore, propolis from one beehive usually consists of exudates from one, two, and rarely three or more dominant plant species (plant precursors of propolis). As a result, propolis types around the world usually coincide with the spread of bees “favorite” plant precursors [3]. Bee’s selectivity is so high, that sometimes plants of foreign species may exclude local flora as a plant precursor.

Despite the unstable composition of globally assessed propolis, its profile of biological activity is usually not so variable [2,4]. Differences were often noted for level of single activity [2,4]. Propolis researchers usually explained this fact by bees’ discrimination of propolis plant precursors: probably, honey bees look for sources with similar physiochemical properties. Moreover, some components of exudates such as terpenes, terpenoids, and polyphenols are suspected to be bee’s attractants [3].

Data on propolis from some of the investigated regions is known, while from others remains unknown or partially investigated. Main unexplored and weakly explored regions are Kyrgyzstan and Kazakhstan. Propolis from Russia (main area of this research) was previously investigated by means of LC-DAD [5,6], LC-MS [5,7,8,9], GC-MS after silylation [10], and some antioxidant assays [5,8]. However, most of these studies investigated only a few antioxidant properties of Russian propolis samples or LC-MS, and antioxidant properties were not the target of the research. Moreover, wide chemical composition screening was performed only via GC-MS after silylation by Isidorov et al. [10]. While GC-MS after sylilation is probably the best method for simultaneous screening of polar and non-polar components, it may fall short for accurate research of polar polyphenols.

The aims of this study were to:

1. investigate chemical profiles of Eurasian propolis samples, including weakly researched, rare samples from Kyrgyzstan and Kazakhstan through ultra-high-performance liquid chromatography–diode array detector–mass spectrometry (UPLC-DAD-MS);
2. evaluate the classical antioxidant properties, which are based on electron donation mechanism, by using DPPH radical scavenging assay, oxygen radical absorbance capacity (ORAC), and ferric reducing antioxidant power (FRAP) assays;
3. assess the relations between the polyphenolic profile of propolis, plant origin, and classical antioxidant activity.

Our direct target of research was aqueous ethanol extract (70:30; ethanol: water; *v*/*v*) due to its wide use in folk medicine and good extracting capacity of polyphenols. UPLC-DAD-MS was chosen as an analytical method because of its adequacy and being an up to date method for polyphenol research in bee products such as propolis [1,2,5]. Antioxidant tests were chosen due to their complementarity.

## 2. Materials and Methods

### 2.1. Chemicals and Reagents

Acetonitrile, formic acid, and water MS grade were obtained from Waters (Milford, CT, USA). Deionized water, gallic acid, and quercetine were purchased from Extrasynthese (Genay, France). DPPH (2,2-diphenyl-1-picrylhydrazyl), TPTZ (complex of 2,4,6-Tri(2-pyridyl)-s-triazine), iron(II) sulfate heptahydrate, and aluminium chloride hexahydrate were obtained from Sigma-Aldrich (Saint Louis, MO, USA). Folin-Ciocalteu reagent, ethanol (analytical grade), and methanol (analytical grade) was purchased from ChemPur (Piekary Śląskie, Poland). OxiSelect™ ORAC Activity Assay was purchased from Cell Biolabs (San Diego, CA, USA). Disodium hydrogen phosphate and sodium chloride was obtained from POCH (Gliwice, Poland).

### 2.2. Sample Preparation

Propolis from the following states was obtained: 11 samples from Russia (regions: Barnaul, Bashikiria, Khabarovsk Krai, Krasnodar Krai, Novosybirsk, Perm, Tomsk and Vologoda Oblast, and Kedrovaja Pad), three samples from Ukraine (Khmelnitsky Village and Tarnopol), and from Kirgizja, Kazahstan (Almastka Oblast), Slovakia (Nova Bana), and Poland (Lubelszczyzna region) were obtained one sample per state. Russian samples were obtained from Professor Valery Isidorov.

Obtained propolis was frozen in liquid nitrogen and crushed in mortar. Freezing and crushing were performed in triplicate (until powdering of propolis). Previously ground research material was extracted by ethanol in water (70:30; *v*/*v*) in proportion with 1.0 g of propolis per 10 mL of solution. Extraction was performed in an ultrasonic bath (Sonorex, Bandelin, Germany). Extraction conditions were set on 40 °C for 45 min and 756 W (90% of ultrasound bath power). Next, extracts were stored at room temperature for 12 h and then filtered through Whattman No. 10 filtrate paper (Cytiva, Marlborough, MA, USA). 

### 2.3. UPLC-DAD-MS

Composition of propolis extracts was analyzed by Waters Acquity UPLC system (Waters, Milford, CT, USA) equipped with PDA 200–500 nm, mass spectrometer Xevo-Q-TOF (Waters, Milford, CT, USA) and column BEH C18 130 Å, (1.7 μm, 2.1 × 150 mm) (Waters, Milford, CT, USA). Analyses were performed according to modified previous methods [11]. A longer column (150 mm compared to previous 100 mm) was used, and eluent gradient was modified. The rest of the parameters of analysis remained the same. Measurements were performed in UV mode and electrospray negative ionization mode. The new elution system consisted of acetonitrile/0.1% solution formic acid in water. The gradient elution program began with 20% acetonitrile and maintained to 3.10 min, then set on 30% in 20.70 min, 31% in 23.30 min and maintained to 25.9 min, 32% in 28.00 min, 33% in 30.50 min, 34% in 36.00 min, 36% in 40.30 min, 40% in 45.50 min, 45% in 47.5 min, 50% in 48.70 min, 75% in 53.0 min, 100% in 56.00 and maintained to 59.00 min, 20% in 60.00 min.

Data was processed using Masslynx 2.0 (Waters, Milford, CT, USA). Single components were identified by comparison of experimental pseudomolecular (precursor) ion mass, mass fragmentation spectra, UV absorption spectra, and retention time to standards and the literature data (articles and metabolite databases).

UV peaks were integrated in range 200–500 nm. For statistical analysis of chemical composition, peaks of UV chromatograms were integrated in the range of 200–500 nm. Area of integrated peaks was calculated as a percentage (%) of combined area of all peaks. 

Moreover, the area of these peaks was used for the relative evaluation of components concentration. Relative concentration was classified as trace (tr), + (low), ++ (average), and abundant (+++). 

### 2.4. Antioxidant and Reducing Activity Determination

Antiradical activity (DPPH Test), reducing activity (FRAP Assay), total phenolic content (TPC), and total flavonoid content (TFC) assays were performed according to previously described methods [12]. Total antioxidant capacity (ORAC measurement) was performed according to Thaipong et al. [13]. All measurements were performed in triplicate. Results of DPPH and TPC were presented as gallic acid equivalents per gram of propolis [mg GAE/g], TFC as quercetin equivalents per gram of propolis [mg QE/g], FRAP as mmol of Fe^2+^ quercetin equivalents per gram of propolis [mmol Fe^2+^/g], and ORAC as mmol of Trolox equivalents per gram of propolis [mmol Trx/g]. For statistical purposes, non-flavonoid polyphenols content [TPC-TFC] was calculated as difference between TPC and TFC.

### 2.5. Statistical Analysis

Statistical analysis was performed in Statistica 13.3 software (StatSoft Power Solutions, Inc./Dell, Round Rock, TX, USA). Analyses include Pearson-correlation tests (between DPPH, FRAP, ORAC, TPC and TFC) and principal component analysis. Principal component analysis was performed in the matrix correlation model. DPPH, FRAP, ORAC, TPC, and TFC were used as variables. All data was normalized before PCA.

Comparison and classification of propolis chemical compositions were performed by cluster analysis based on Pearson correlation and complete linkage clustering by Statistica 13.3 software (StatSoft Power Solutions, Inc./Dell, Round Rock, TX, USA). Matrix consisted of normalized UV peak areas (see Section 2.3).

## 3. Results and Discussion

### 3.1. Profile of Polyphenols and Classification of Eurasian Propolis

**Figure 1:**
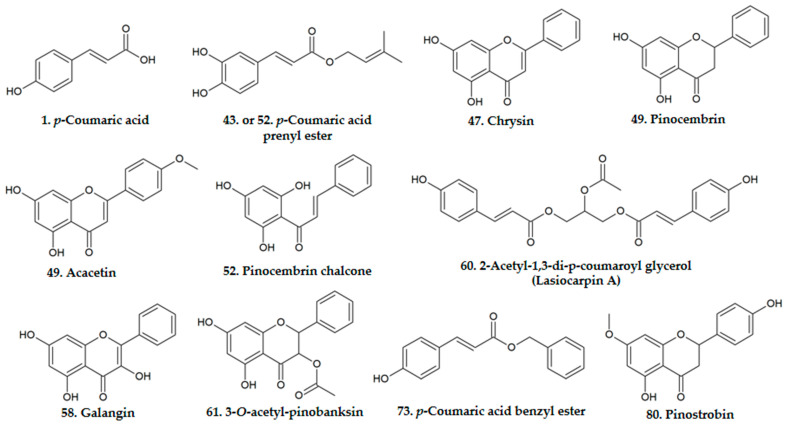
Chemical composition of Eurasian propolis.

Results of UPLC-DAD-MS are shown in Table 1 and Table 2. Main components of Eurasian propolis are shown in Figure 1. Eighty-six substances were identified or tentatively identified. Identification included components identified by comparison with standards or substances which MS, and UV spectra are well established in the literature. Moreover, these components were often isolated from different propolis or its precursors by researchers. 

Some substances were classified as tentatively identified. This included geometric structure (regio- and stereoisomers, e.g., caffeic acids prenyl or isoprenyl esters) and substitution position (e.g., dimethyl quercetin and some phenolic acids glycerides). Moreover, some components were also tentatively identified due to lack of sufficient UV and mass data or trace concentration.

Generally, five main chemical groups were observed: flavonoids, free cinnamic acids, cinnamic acids monoesters, phenolic acids glycerides, and other components. Flavonoids aglycones were main components of most samples (10), but different aglycones dominated in different propolises. The rest of the samples were a mix of flavonoids and other polyphenols. However, among them, only SO (propolis from Saratov Oblast, Russia) contained low peaks of flavonoid in comparison to rest polyphenols. It was composed of free cinnamic acids, cinnamic acids monoesters, and phenolic acids glycerides.

The flavonoids group was the most extensive group of components and contained 43 substances. Moreover, peaks of these components were often dominant. The main flavonoids were chrysin, pinocembrin, acacetin, pinocembrin chalcone, galangin, 3-*O*-acetyl-pinobanksin, and pinostrobin. Among them, only chrysin was present in all of the samples, albeit in one as trace. Most of the flavonoids were aglycones, and only one substance was tentatively identified as glucoside (Apigenin-7-*O*-glucoside). Usually propolis exhibited the presence of a significant amount of flavonoid aglycones and absence of glucosides. Authors connected this fact with the protective function of plant exudates [14]. In terms of plant origin, flavonoid aglycones or absence thereof are usually specific markers of different propolis precursors around the world [15,16].

Five substances were identified in the free cinnamic acids group. The dominant peak belonged to *p*-coumaric acid, while rest of the substances (caffeic, ferulic, isoferulic acids, and cinnamic acids) exhibited trace, low, or intermediate peaks. However, at least one free cinnamic acid was observed in all samples. These components are usually present in most propolis samples [3,17], but for this reason, free cinnamic acids are usually non-specific markers of propolis plant precursors, with some exceptions such as C-prenylated *p*-coumaric acids [2,18].

Phenolic acids monoesters group was composed of 17 substances. Among them, the dominant peaks were of different isomers of caffeic acid prenyl or isoprenyl esters and benzyl esters of *p*-coumaric and caffeic acid. Some of the cinnamic acids monoesters may be markers of propolis plant origin [15,16].

The phenolic acids glycerides group consisted of 17 components. The dominant peaks were observed for 2-acetyl-1,3-di-*p*-coumaroylglycerol. Other considerable compounds were 2-acetyl-1-*p*-coumaroyl-3-feruloylglycerol and 2-acetyl-1,3-di-feruloylglycerol. Usually, this type of component—glycerol substituted by acetic acid and two cinnamic acids (*p*-coumaric, ferulic/isoferulic, caffeic and cinnamic acids)—is most often found in propolis [10,11,19]. Non acetylated derivatives and mono or three cinnamics acids substituted form are rarer in propolis [10,11,19]. Potentially, this fact may be connected with them being at the end of the metabolic pathway in plants (full substitution of glycerol) and/or protective function of acetylation [11]. Generally these components are considerably specific markers of *P. tremula* exudates [10,11,19], but these compounds are also present in others plants [12].

The last group included only four components, and the strongest peak was benzoic acid. Benzoic acid is suspected to be a product of benzoic acid esters degradation (especially benzyl benzoate, which is often a major component of propolis essential oils) [11].

**Figure 2:**
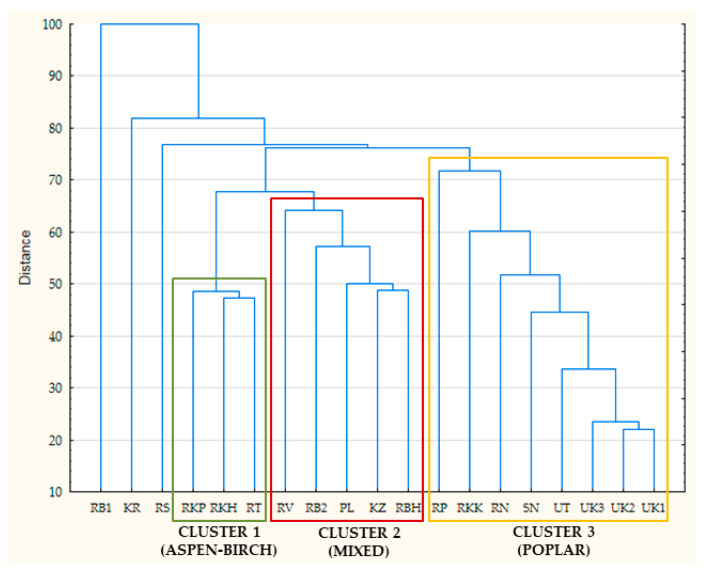
Classification of Eurasian propolis.

Research on Eurasian propolis showed that it derives mainly from poplar, aspen [10,11,39], or birch [10,39], or has mixed origin [10,11,39]. Hierarchical fuzzy clustering analysis allowed the grouping of samples in the three major clusters (Figure 2). The clusters were directly connected with plant origin.

Cluster 3 consisted of propolis, which may be classified as poplar (or more accurately black poplar) type. Presence of other plant precursor markers, mainly aspen and birch, was low, or there were just traces. The main poplar markers in Cluster 3 were flavonoid aglycones (chrysin, pinocembrin, 3-*O*-acetyl-pinobanksin, and galangin [10,11]). These components are usually specific markers of black poplar origin determined by different analytical methods [10,11,24], but sometimes others components may be better markers due to variability of *P. nigra* chemotypes [10,11]. They include other flavonoid aglycones (e.g., pinocembrin and pinostrobin chalcones [11]), caffeic acid esters [10,11], or high amount of free cinnamic acids (especially isoferulic acid) [10,11]. Our research sample from Kyrgyzstan (KR) has shown a typical poplar profile. It contained typical poplar markers (chrysin, pinocembrin, 3-*O*-acetyl-pinobanksin, and galangin); however, there were also its own specific components such as pinocembrin chalcone and significant peaks of caffeic acid prenyl or isoprenyl esters present. Moreover, *p*-coumaric (and its esters), ferulic, and isoferulic acid were absent. Therefore, despite the evident poplar profile (presence of *P. nigra* flavonoids), it was grouped outside Cluster 3.

In the case of aspen propolis, wide spread of *P. tremula* suggested it as the main plant precursor of aspen propolis in Eurasian area. Markers of this type include different cinnamic acids glycerides, especially 2-acetyl-1,3-di-*p*-coumaroyl glycerol [10,11]. This component, known also as lasiocarpin A, was previously isolated from *P. lasiocarpa* buds by Asakawa et al. [40]. Apart from cinnamic acids glycerides, aspen buds contain low amounts of flavonoids, but typical poplar markers (chrysin, pinocembrin, 3-*O*-acetyl-pinobanksin, and galangin) are absent [10,11]. In the case of phenolic acids monoesters, some of these components are present in poplars and aspens (*p*-coumaric and caffeic acids benzyl ester and *p*-coumaric acids ferulic ester) [10,11], while others are characteristic for poplars (for example CAPE, different isomers of caffeic acids prenyl and isoprenyl esters) [10,11].

Birch propolis contains mainly sesquiterpenoids (birkenal, different isomers of caryophyllene alcohols, and acetates) [10] and some flavonoid aglycones (sakuranetin and pectolinaringenin) [10] as markers. Flavonoid aglycones were mainly components of *Betula pubescens* (white birch), while *B. pendula* (silver birch) contained trace amounts of these components [20]. In our previous research [11], sakurantetin was also present in both poplars and aspens (higher concentration in aspens), while other research showed absence or trace amounts in aspens and *B. pendula* [10,41], low concentration in some *P. nigra,* and high concentration in *B. pubescens*, respectively [10]. The pectolinaringenin situation is also complicated in researched propolises. Instead of pectolinaringenin in propolis, we found its isomer ermanin (kaempferol-3,4’-dimethyl ether, M = 314), which was isolated from *B. pendula* buds [42]. Pectolinaringenin was excluded due to a different UV spectrum. However, we find more common components of some *B. pubescens* buds chemotypes [41] such as acacetin (M = 284) and kaempferide (M = 300). Among these components, kaempferide was also observed in a considerable amount in *P. tremula* buds and less in *P. nigra* [11].

According to known information about poplars, aspens, and birch [10,20,41,42], propolis grouped in Cluster 1 (RPK, RKH, RT) may be classified as aspen–birch. The main aspen markers of Cluster 1 were lasiocarpin A, *p*-coumaric acid benzyl ester, *p*-coumaric, and ferulic acid. Birch markers of this cluster included acacetin and ermanin. There is also observed common birch–aspen marker—sakuranetin. Stronger peaks of aspen markers suggest domination of *P. tremula* exudates in the cluster rather than *Betula*.

Cluster 2 contained markers of aspen, poplar, and birch with unresolved dominance of precursors in most cases. Therefore, they are classified as mixed (aspen–birch–poplar propolis). It was worth adding that RV exhibited strong dominance of aspen markers (mainly 2-acetyl-1,3-di-*p*-coumaroyl glycerol), but due to strong peaks of kaempferide, RV is more similar to Cluster 2 than Cluster 1. Last two samples (RB1 and RS) were mixed propolis. However, the differences between them and rest of the samples excluded them from main clusters. RB1 exhibited a significant amount of caffeic acid esters, which was not observed in Cluster 2. The last sample (RS) is aspen–poplar propolis with dominance of aspen markers and without specific birch markers (ermanin).

Apart from known plant precursors such as *P. nigra* and *P. tremula*, other *Populus* species also produce sticky exudates and may be propolis precursors’ sources. Possible plant sources include many *Populus* species (ex. *P. laurifolia* or *P. maximowiczii*). Moreover, different *Populus* trees are known for their ease in forming hybrid species [43], and compositions of these species buds exudates are usually unknown. As a result, accurate prediction of plant precursors from poplar species is difficult. However, general classification of propolis is possible.

### 3.2. Antioxidant Properties and Phenolic Content of Eurasian Propolis

Results of colorimetric assays (TPC, TFC, DPPH, FRAP, and ORAC) assays were shown in Table 3. Propolis was also divided into plant origin groups according to results of hierarchical fuzzy clustering analysis and chemical composition (described in the last section). The most abundant amount of polyphenols was observed in Ukrainian propolis from Tarnopol (UT, 145.24 mg GAE/g) while the lowest was in Kirgisian propolis (KR, 30.28 mg GAE/g). UT contained also the highest amount of flavonoids (82.71 mg GAE/g) and the lowest TFC was shown for Russian propolis from Saratov Oblast (10.45 mg GAE/g). Moreover, correlation between TFC and plant origin (*p* = 0.016) and the highest amount of flavonoid was observed for P group (Poplar origin).

DPPH test is used to evaluate the radical-scavenging activity—the ability to “catch free radicals”. Generally, components with high radical scavenging activity work as electron donors and can inactivate free radicals by different mechanisms (e.g., generation less reactive form of radical) and biological targets of oxidative stress are to be protected [44,45]. In our research, the highest radical scavenging activity was observed for samples from Barnaul (RB1, 64.47 mg GAE/g) and the lowest for KR (8.83 mg GAE/g). Statistical analysis showed that only [TPC-TFC] correlated with radical scavenging activity (*p* = 0.011). This suggests that flavonoids did not exhibit any role in radical scavenging activity. The structure of propolis flavonoids and other phenols may explain this observation. Propolis contains mainly flavonoid aglycones with B rings without hydroxyl groups (OH) or with OH groups in A, B, and C rings blocked by methyl groups (-CH_3_) or ester groups (-COR). It is known that free OH groups in these positions exhibit an important role in stabilization of flavonoid molecules after “attack of free radical” [44,45]. Phenolic OH groups (especially catechol rings) are critical determinants of the electron-/H-atom-donating activity [44,45]. Therefore, their amount and position in aryl rings is crucial for radical scavenging activity. Apart from flavonoids, cinnamics acids, and their esters and glycerides are mainly composed of cinnamic acid derivatives substituted by OH groups.

Comparison of caffeic acid phenethyl ester (CAPE) and chrysin, which are common propolis components, showed that CAPE exhibited better radical scavenging activity than chrysin due to better delocalization of electrons in radical form of CAPE [46]. According to the results, CAPE was more sensitive to free radical attack and exhibited higher radical scavenging activity [46]. However, it is worth adding that flavonoids with less OH groups may incorporate into cell membranes and protect them from free radical attacks [47]. As a result, more hydrophobic flavonoid aglycones also may play an important role in antioxidant activity. Apart from comparison of all samples, statistical analyses were also performed inside the propolis group. They were selected according to plant origin (see Table 3). Due to the amount of samples, three significant groups were selected. The first was poplar propolis group (P, n = 9) and aspen–birch–poplars (A-B-P, n = 6) and aspen–birch (A-B, n = 3). These results were more complex and depended on the tested group. While, in group A–B, DPPH was correlated only with TPC (*p* = 0.037), in group P correlations were observed between DPPH and TPC (*p* = 0.015), TFC (*p* = 0.06) and ORAC (*p* < 0.0005). For the A–B–P group, there was an observed correlation between DPPH and TPC (*p* = 0.007), TFC (*p* = 0.016), and [TPC-TFC] (*p* = 0.003) but not with ORAC. As a result, different polyphenols were considerable markers of radical scavenging activity. Distinguished from “global calculation”, TFC was a considerable marker of antioxidant activity due to a higher amount of flavonoids than non-flavonoid polyphenols in P group. But for A-B-P, TFC exhibited less significant impact on DPPH than TPC and [TPC-TFC]. This suggests that DPPH correlates with an amount of flavonoids and non-flavonoids polyphenols, but non-flavonoids polyphenols exhibited higher impact on radical scavenging activity of this group.

Distinguished from DPPH, FRAP (ferric reducing antioxidant power assay) test and ORAC (oxygen radical absorbance capacity) is used to evaluate antioxidant capacity. In our research, the strongest FRAP activity was exhibited by RV (1.17 ± 0.06 mmol Fe^2+^/g) and the lowest by RT (0.06 ± 0.01 mmol Fe^2+^/g). FRAP activity was not correlated with TPC nor TFC among all propolis samples. Moreover, analysis inside groups (P and A-B-P) also showed this same result. This observation may suggest that reducing activity is a result of interaction between specific components. This hypothesis may be supported by high activity of mixed propolises (A-B-P). Composition of these propolises was the most complex among the samples, and potentially in FRAP’s activity, it may play a role in non-polyphenols components such as terpenoids. On the other hand, inside group A-B, FRAP was correlated with TPC (*p* = 0.014), which suggests connection between all phenols and FRAP activity.

ORAC is a test that evaluates how many free radicals can be deactivated by tested samples. Therefore, ORAC was correlated only with TPC (*p* = 0.034). Analysis among plant origin groups exhibited correlation between ORAC and TPC (*p* = 0.036), TFC (*p* = 0.039), and DPPH, but not with [TPC-TFC] in group P. This suggested that ORAC values were determined mainly by the amount of flavonoids according to Pearson correlation factor. Same non-flavonoid samples exhibited weak or no impact. However, no correlation with ORAC was observed inside groups A-B-P and A-B. This suggests that in all of the propolises, all polyphenols exhibit impact on ORAC results, but inside the poplar group, a considerable factor was only flavonoids. Results for A-B-P and A-B groups suggest that the ORAC test could be affected also by non-polyphenols components. Potentially, this group may be sesquiterpenoids, which are present in highly significant amounts in birch exudates [10,41]. It is worth adding that difference between samples for the ORAC test was quite less pronounced than in other colorimetric assays.

In summary, poplar propolis exhibited the most variable content of polyphenols and antioxidant activity (DPPH and FRAP assays), while mixed aspen-birch-poplar propolis usually had strong antioxidant activity (DPPH and FRAP assays).

## 4. Conclusions

The present research shows that Eurasian propolis is a rich source of polyphenols and potent antioxidant agent (in classical terms of electron donation mechanism). Different assays of antioxidant activity were correlated with flavonoids, total polyphenols content, or the content of polyphenols other than flavonoids. Results may suggest complex dependencies between propolis plant origin and its activity. Investigated propolis exhibited typical poplar or mixed plant origin (aspen-birch, aspen-poplar and aspen-birch-poplar).

Flavonoids aglycones were main components of most samples (10), but different aglycones dominated in different propolises, while the rest of the samples were a mix of flavonoids and other polyphenols. Only one sample consisted mostly of non-flavonoid polyphenols. Chemical markers have proven that the majority of samples were either poplar or poplar with admixtures of other species in origin. Samples from rare research areas exhibited poplar (Kyrgyzstan) and mixed origin (aspen-birch-poplar, Kazakhstan).

All of the investigated samples exhibited antioxidant and radical scavenging activity, albeit to different degrees. The results pointed out DPPH results correlating with the amount of both flavonoids and non-flavonoids polyphenols, but non-flavonoids polyphenols played a more important role in activity of the samples. FRAP results, on the other hand, seem to be correlated with general polyphenol content. The differences between samples for the ORAC test were significantly less pronounced than in other colorimetric assays and seemed to be correlated to both flavonoids and non-flavonoids polyphenols as well as non-phenolic compounds.

## Figures and Tables

**Table 1:** UPLC-DAD-MS analysis of Eurasian propolis.

**No.**	**Component**	**RT**	**UV λ_max_ (nm)**	**[M − H]^−^**	**Mass Fragments**	**Ref**
1	* Caffeoylglycerol ^b,c^	1.88	**324**, 298sh, 242	253	179, 161, 135	[20]
2	*p*-Hydroxy benzoic acid ^a,b,c^	2.13	**256**	137	-	[21]
3	Caffeic acid ^a,b,c^	2.17	**324**, 298sh, 242	179	135	[10,22,23,24,25,26,27]
4	* p-Coumaroylglycerol ^b,c^	2.63	**310**, 300sh, 229	237	163, 145, 119	[11,19,20]
5	*p*-Coumaric acid ^a,b,c^	3.25	**310**, 300sh, 229	163	119	[10,22,23,24,25,26,27]
7	Vanilline ^a,b,c^	3.42	**310**, 280, 231	151	-	[11,22,27]
8	Ferulic acid ^a,b,c^	3.70	**324**, 298sh, 236	193	149, 134	[11,19,20]
9	Isoferulic acid ^a,b,c^	4.11	**323**, 295sh, 221	193	149, 134	[11,19,20]
10	Benzoic acid ^a,b,c^	5.97	281sh, 274sh, **236**	121	103, 77	[11,22,27]
11	* Ferulic acid derivate	6.82	**326**, 298sh, 236	389	193, 175, 148, 134	-
12	Acetyl-*p*-coumraoylglycerol ^b,c^	7.40	**311**	279	219, 163, 145, 119	[11,19,20]
13	* Apigenin-7-*O*-glucoside ^b,c^	8.07	315sh, **265**	431	268, 239, 195	[24]
14	3,4-Dimethylcaffeic acid ^b,c^	8.52	**322**, 294sh, 236	207	163, 133	[11,23,24]
15	Luteolin ^a,b,c^	11.30	349, 290sh, 270sh, 254, 227sh	285	213, 151	[11,23,24]
16	Quercetin ^a,b,c^	11.43	368, 293sh, 270sh, **256**	301	271, 151, 121, 107	[21,24]
17	Pinobanksin-5-methyl ether ^b,c^	12.32	322sh, **288**, 228	285	267, 252, 239, 224, 208, 195, 180, 165, 152,136	[23,24,26]
18	Cinnamic acid ^a,b,c^	12.94	**277**	147	103	[11,23,24,27]
19	Quercetin-3-methyl ether ^b,c^	13.68	355, 293sh, 268sh, **255**	315	300, 271, 243	[11,23]
20	* 1-Caffeoyl-3-*p*-coumaroyl glycerol ^c^	15.23	**315**, 298sh, 235	399	253,235, 219, 179, 163, 135, 119	[10,11,24]
21	Naringenin ^a,b,c^	15.83	330sh, **290**, 230	271	269, 227, 177, 165, 151, 119, 107, 93	[24,28]
22	Pinobanksin ^a,b,c^	16.30	332sh, **292**, 229	271	253, 225, 209, 185, 151, 107	[10,11,23,24]
23	Apigenin ^a,b,c^	16.40	**338**, 290sh, 268, 226	269	227, 181, 151, 149, 117, 107	[11,23,24]
24	* Caffeoyl-feruloylglycerol ^b,c^	16.12	326, 298sh, 240	429	267, 253, 249, 235, 193, 179, 149, 135, 134	[11,19,20]
25	Chrysin-5-methyl ether ^b,c^	16.80	314sh, **264**, 247sh	267	252, 242, 180	[11,25]
26	Kaempferol ^a,b,c^	17.48	366, 266, 248sh	285	-	[10,11,24,27]
27	Isorhamnetin ^a,b,c^	19.11	371, 298sh, 268sh, **255**, 233sh	315	300, 151	[10,24,26,27]
28	* Quercetin-methyl ether ^b,c^	19.57	371, 298sh, 268sh, **255**, 233sh	315	300,165, 151, 193, 121, 109	[3,5]
29	Luteolin-5-methyl ether ^b,c^	20.15	350, 298sh, **266**, 232sh	299	284, 255, 227, 211	[11,23,25]
30	* 1,3-Di-*p*-coumaroylglycerol ^b,c^	20.25	**310**, 300sh, 233	383	237, 219, 163, 145, 119	[10,11]
31	Quercetine-5,7-dimethyl ether ^b,c^	20.71	356, 296sh, 269sh, **255**	329	314, 299, 285, 271, 257, 243, 227	[3,5,7]
32	* *p*-Coumaroyl-feruloylglycerol ^b,c^	21.37	**316**, 298sh, 233	413	249, 237, 219, 179, 163, 149, 134, 119	[11,24,29]
33	* di-1,3-Feruloylglycerol ^b,c^	21.95	**323**, 298sh	443	249, 193	[10,11,24]
34	2-Acetyo-1,3-di-caffeoylglycerol ^b,c^	22.57	**328**, 298sh, 244	457	397, 295, 235, 179, 163, 161, 135	[11,24,29]
35	*β*-Styrylacrilic acid (cinnamylideneacetic acid) ^b,c^	23.82	**311**, 240sh	173	-	[23]
36	Galangin-5-methyl ether ^b,c^	25.42	352, 300sh, **261**, 240sh	283	268, 239, 211	[23,26]
37	Pinobanksin-3-*O*-acetyl-5-methyl-ether ^b,c^	25.60	**289**	327	285, 267, 252, 224	[23,26]
38	* Caffeic acid butenic or isobutenic ester ^b,c^	24.73	**326**, 298sh, 248	233	179, 161, 135	[11]
**No.**	**Component**	**RT**	**UV λ_max_ (nm)**	**[M − H]^−^**	**Mass Fragments**	**Ref**
39	Rhamnetin ^a,b,c^	25.92	354, 298sh, 268sh, **255**	315	300,165, 151, 193, 121, 109	[11,24]
40	Quercetin-3,3-dimethyl ether ^b,c^	27.22	356, 292sh, 268sh, **256**	329	314, 299, 271, 243, 227	[11,23]
41	2-Acetyl-1-caffeoyl-3-*p*-coumaroylglycerol ^b,c^	29.23	**316**, 299sh 235	441	381, 295, 235, 179, 163, 135, 119	[10,11,19,24]
42	* Quercetin-dimethyl ether ^b,c^	29.46	356, 292sh, 268sh, **256**	329	300,165, 151, 193, 121, 109	[11,23,24,25,26,27]
43	* Caffeic acid butyl or isobutyl ester ^b,c^	30.20	**326**, 298sh, 242	235	179, 161, 135	[11]
44	2-Acetyl-3-caffeoyl-1-feruloylglycerol ^b,c^	30.28	**328**, 300sh, 244	471	411, 295, 235, 193, 179, 149, 135	[11,19,26]
45	Quercetin-3,7-dimethyl ether ^b,c^	30.63	356, 292sh, 268sh, **256**	329	300,165, 151, 193, 121, 109	[21,24]
46	* Caffeic acid prenyl or isoprenyl ester 1 ^b,c^	31.92	**326**, 298sh, 246	247	179, 161, 135	[23,24,25,26,27]
47	Chrysin ^a,b,c^	32.11	314sh, **268**, 246sh	253	209, 181, 167, 165, 151, 107, 145, 143, 119	[10,11,23,24,25,26,27]
48	Sakuranetin ^b,c^	35.16	328sh, **291**, 270sh, 247sh	285	165, 119	[24,27]
49	Pinocembrin ^a,b,c^	33.68	330sh, **290**, 235	255	213, 187,151, 145, 136	[11,23,26,27]
50	Acacetin ^a,b,c^	34.08	**335**, 299sh, 268, 228	283	268, 239, 211, 183, 151, 107	[30]
51	* Caffeic acid prenyl or isoprenyl ester 2 ^b,c^	34.10	**326**, 298sh, 246	247	179, 161, 135	[23,24,25,27]
52	Pinocembrin chalcone ^b,c^	34.14	**345**	255	213, 151, 101	[10,31]
53	* Caffeic acid prenyl or isoprenyl derivate 1 ^b,c^	34.21	**326**, 298sh, 246	247	179, 161, 135	[11,23,24,25,26]
54	Caffeic acid benzyl ester ^b,c^	34.62	**328**, 298sh, 244	269	178, 161, 134	[11,23,24,25,26]
55	* Caffeic acid prenyl or isoprenyl derivate 2 ^b,c^	34.58	**326**, 298sh, 246	247	179, 161, 135	[11,23,24,25,26]
56	* Flavonoid ^b,c^	33.62	332sh, **291**, 232	285	255, 145, 139, 124	-
57	Genkwanin ^a,b,c^	35.64	337, **267**, 242sh	283	268, 239, 211, 183, 171, 151, 117, 107	[32]
58	Galangin ^a,b,c^	36.07	360, 314sh, 290sh, **266**, 240sh	269	227, 197, 183, 151	[10,11,21,23,24,25]
59	Kaempferide (Kaempferol 4’-methyl ether) ^b,c^	37.31	366, 322sh, 292sh, **266**, 250sh	299	284, 255, 227, 164, 151	[21,27]
60	2-Acetyl-1,3-di-*p*-coumaroylglycerol ^b,c^	37.55	360sh, **312**, 232	425	365, 321, 215, 163, 119	[10,19,24]
61	3-*O*-Acetyl-pinobanksin ^b,c^	37.82	332sh, **294**, 238	313	271, 253, 209, 181, 165, 143, 151, 107	[10,11,23,24]
62	* Quercetin-dimethyl ether ^b,c^	38.33	**370**, 268sh, 255	329	314, 299, 284, 271	[11,21,23,26,27]
63	* 2-Acetyl-3-*p*-coumaroyl-1-feruloylglycerol ^b,c^	38.96	**318**, 299sh 235	455	395, 351, 193, 163, 149, 119	[10,11,29]
64	* Metoxychrysin ^b,c^	39.27	340sh, 310sh, **266**, 245sh	283	268, 239, 211, 195	[11,23,26]
65	3-Acetyl-1,2-di-*p*-coumaroylglycerol ^b,c^	40.09	**312**, 300sh, 238	425	365, 163	[11,20]
66	Caffeic acid phenethyl ester (CAPE) ^b,c^	40.15	321, 300sh, 264 232	283	179, 161, 119	[11,23,26]
67	2-Acetyl-1,3-di-feruloyl glycerol ^b,c^	40.37	**328**, 298sh, 243	485	425, 249, 230, 193, 175, 149, 134	[10,11,29]
68	Ermanin (Kaempferol 3,4’-dimethyl ether) ^b,c^	41.05	348, **267**, 246sh	313	298, 283, 269, 255, 227, 211, 183, 155, 117	[33]
69	* Caffeic acid pentyl or isopentyl ester 1 ^b,c^	41.90	**326**, 298sh, 247	249	179, 161, 134	[34]
70	Ayanin (3,7,4’-Trimethylquercetin) ^b,c^	42.53	**343**, 271, 248sh	343	328, 313, 298, 285, 270, 255, 242, 214, 186, 163, 145, 129, 113	[35]
71	* *p*-Coumaric acid prenyl ester 1 ^b,c^	45.42	**311**, 299sh, 245sh	233	163, 145, 119	[11,23,25,26]
72	* *p*-Coumaric acid prenyl ester 2 ^b,c^	45.42	**311**, 299sh, 245sh	233	163, 145, 119	[11,23,25,26]
73	*p*-Coumaric acid benzyl ester ^b,c^	45.80	**312**, 298sh, 244sh	253	162, 145, 118	[10,11,23,26]
74	* Ferulic or isoferulic acid benzyl ester ^b,c^	47.13	**326**, 298sh	283	-	[10,11,36]
75	Caffeic acid cinnamic ester ^b,c^	48.04	**326**, 300sh, 243	295	178, 163, 134, 92	[11,23,24]
76	3-*O*-propyl-pinobanksin ^b,c^	48.58	329sh, **294**, 234	327	271, 253, 225, 209, 181, 165, 143	[11,23,24]
77	*p*-Coumaric acid phenethyl ester ^b,c^	49.21	**312**, 300sh, 242sh	267	163, 145, 119	[11,25]
**No.**	**Component**	**RT**	**UV λ_max_ (nm)**	**[M − H]^−^**	**Mass Fragments**	**Rf**
78	Pinostrobin chalcone ^b,c^	49.67	**345**, 309sh, 267	269	254, 226, 198, 171, 165, 136, 122	[11,24]
79	Tectochrysin (Chrysin-7-methyl ether) ^b,c^	51.23	310sh, **268**	267 ^iw^	-	[37]
80	Pinostrobin (Pinocembrin-5-methyl ether) ^a,b,c^	51.40	328sh, **290**, 248sh	269 ^iw^	-	[11,12,28]
81	3-*O*-butyl or isobutyl pinobanksin ^b,c^	51.65	320sh, **293**, 240sh	341	271, 253, 209, 181, 165, 151, 107	[23,26]
82	Galangin-7-methyl ether ^b,c^	52.21	353, **268**	283 ^iw^	-	[38]
83	3-*O*-pentyl or isopentyl pinobanksin 1 ^b,c^	53.07	332sh, **293**, 242	355	253, 209, 181, 165, 143, 107, 101	[23,24,26]
84	3-*O*-pentyl or isopentyl pinobanksin 2 ^b,c^	53.24	320sh, **295**, 250	353	271, 253, 209, 181, 165, 151, 107	[23,24,26]
85	3-*O*-hexyl-pinobanksin ^b,c^	54.09	**282**	369	271, 253, 209, 151, 143	[23,24,26]
86	* Metoxycinnamic acid cinnamyl ester ^b,c^	54.26	**280**	293	-	[23]

^a^ component identified by comparison with standard; ^b^ component identified by comparison with literature; ^c^ component identified by prediction of mass fragment and UV spectrum; * component tentatively identified; ^iw^ component produces low or trace amount of ions, sh—shoulder peak. The bold signifies the main ion.

**Table 2:** Relatively concentration of components in UPLC-DAD analysis.

**No.**	**Component**	**UK1**	**UK2**	**UK3**	**UT**	**RBH**	**RB1**	**RB2**	**RKH**	**RKK**	**RKP**	**RN**	**RP**	**RS**	**RT**	**RV**	**KR**	**KZ**	**SL**	**PL**
1	* Caffeoyl-glycerol	tr	tr	tr	tr	tr	tr	-	tr	tr	-	tr	tr	tr	tr	-	tr	tr	tr	tr
2	*p*-Hydroxy benzoic acid	tr	tr	tr	tr	tr	-	tr	tr	tr	tr	tr	-	tr	tr	tr	-	tr	tr	tr
3	Caffeic acid	+	+	+	+	+	+	+	+	+	tr	+	+	+	tr	tr	+	+	+	tr
4	*p*-Coumaroylglycerol	-	-	-	-	tr	tr	tr	tr	-	-	tr	-	tr	tr	tr	-	tr	tr	tr
5	*p*-Coumaric acid	+	+	+	+	++	++	++	++	+	+	+	tr	++	+++	++	-	+	+	++
7	Vanilline	tr	-	-	-	tr	tr	tr	tr	-	+	tr	-	tr	+	+	-	tr	tr	+
8	Ferulic acid	+	+	+	+	tr	+	+	++	+	+	+	tr	++	++	+	-	+	+	+
9	Isoferulic acid	+	+	+	+	+	+	+	tr	+	+	+	+	tr	tr	tr	-	tr	tr	tr
10	Benzoic acid	+	tr	tr	tr	+	+	+	+	tr	+	tr	tr	++	+	+	-	+	tr	++
11	* Ferulic acid derivate	tr	tr	tr	tr	tr	tr	tr	tr	-	-	tr	-	tr	tr	tr	-	tr	tr	tr
12	Acetyl-*p*-coumaroylglycerol	tr	-	-	-	tr	tr	tr	tr	tr	-	-	-	tr	-	tr	-	tr	tr	tr
13	3,4-Dimethylcaffeic acid (DMCA)	tr	tr	tr	tr	+	+	+	-	+	-	tr	+	-	-	tr	-	tr	tr	tr
14	* Apigenin-*O*-glucoside	tr	tr	tr	tr	-	-	-	-	tr	-	tr	-	-	-	-	-	tr	tr	-
15	Luteolin	tr	tr	tr	tr	tr	tr	-	tr	-	-	tr	tr	tr	-	tr	-	tr	tr	-
16	Quercetin	tr	+	+	+	tr	tr	tr	tr	tr	tr	tr	tr	tr	-	tr	+	tr	tr	tr
17	Pinobanksin-5-methyl ether	+	+	+	+	tr	+	-	tr	tr	-	tr	-	tr	-	-	-	tr	+	tr
18	Cinnamic acid	tr	-	-	-	tr	-	tr	tr	tr	Tr	-	-	tr	-	tr	-	-	tr	tr
19	Quercetin-3-methyl ether	tr	tr	tr	tr	tr		tr	tr	tr	-	tr	tr	tr	tr	-	+	tr	+	tr
20	* 1-Caffeoyl-3-*p*-coumaroyl glycerol	-	-	-	-	tr	tr	tr	tr	-	-	tr	-	tr	tr	tr	-	tr	-	tr
21	Naringenine	tr	tr	tr	tr	tr	tr	tr	tr	tr	-	tr	tr	tr	tr	tr	-	tr	-	tr
22	Pinobanksin	+	+	+	+	tr	+	tr	tr	+	tr	+	+	tr	tr	tr	+	+	+	tr
23	Apigenin	+	tr	tr	tr	+	tr	+	+	+	+	tr	tr	tr	+	+	+	+	+	+
24	Caffeoyl-feruloylglycerol	-	-	-	-	-	-	tr	tr	-	-	-	-	tr	tr	tr	-	tr	-	-
25	Chrysin-5-methyle ether	tr	tr	tr	tr	-	-	-	-	tr	-	tr	-	-	-	-	-	tr	tr	tr
**No.**	**Component**	**UK1**	**UK2**	**UK3**	**UT**	**RBH**	**RB1**	**RB2**	**RKH**	**RKK**	**RKP**	**RN**	**RP**	**RS**	**RT**	**RV**	**KR**	**KZ**	**SL**	**PL**
26	Kempferol	+	+	+	+	+	tr	tr	+	tr	+	+	tr	tr	+	+	+	+	+	+
27	Isorhamnetin	tr	tr	tr	tr	+	tr	tr	tr	tr	+	tr	tr	tr	+	+	-	+	tr	tr
28	Quercetin-methyl ether	tr	-	-	-	tr	-	tr	-	tr	-	tr	tr	tr	tr	tr	-	tr	tr	
29	Luteolin-5-methyl ether	+	tr	tr	tr	tr	-	tr	+	+	tr	+	tr	tr	+	+	+	+	+	tr
30	** 1,3-Di-*p*-coumaroylglycerol	tr	tr	tr	tr	Tr	+	+	+	tr	tr	tr	-	+	+	tr	-	tr	tr	tr
31	Quercetine-5,7-dimethyl ether	tr	tr	tr	tr	tr	-	tr	tr	tr	tr	tr	tr	tr	+	+	tr	+	tr	tr
32	*p*-Coumaroyl-feruloylglycerol	-	tr	tr	tr	tr	tr	tr	tr	-	tr	tr	tr	tr	tr	tr	-	tr	-	-
33	Di-feruloyoglicerol	tr	-	-	-	tr	tr	tr	tr	-	tr	tr	-	tr	-	tr	-	tr	tr	-
34	2-Acetyo-1,3-di-caffeoylglycerol	tr	tr	tr	tr	tr	tr	tr	tr	tr	tr	tr	-	tr	-	tr	-	tr	tr	tr
35	β-styrylacrilic acid	tr	tr	tr	tr	-		-	-	-	-	tr	-	-	-	-	-	-	tr	tr
36	Galangin-5-methyl ether	tr	tr	tr	tr	tr	tr	-	-	tr	-	tr	-	-	-	tr	-	tr	tr	
37	Pinobanksin-3-*O*-acetyl-5-methyl-ether	tr	tr	tr	tr	tr	-	-	-	tr	-	tr	-	-	-	tr	-	tr	tr	-
38	* Caffeic acid butenic or isobutenic ester ^b,c^	-	-	-	-	-	-	-	-	-	-	-	-	-	-	-	-	tr	-	-
39	Rhamnetin	tr	+	+	+	tr	tr	tr	tr	tr	-	tr	-	tr	tr	tr	-	tr	+	tr
40	Quercetin-dimethyl ether	-	tr	tr	tr	tr	tr	tr	tr	-	tr	tr	tr	tr	tr	tr	-	tr	tr	tr
41	2-Acetyl-1-caffeoyl-3-*p*-coumaroylglycerol	tr	tr	tr	tr	tr	tr	tr	+	tr	-	tr	-	+	tr	tr	-	tr	tr	tr
42	Quercetin-dimethyl ether	tr	tr	tr	tr	tr	-	tr	tr	tr	-	tr	tr	tr	tr	-	-	tr	+	tr
43	Caffeic acid butyl or isobutyl ester	-	-	-	-	-	-	tr	-	tr	-	tr	+	-	-	-	-	tr	-	-
44	2-Acetyl-3-caffeoyl-1-feruloylglycerol	tr	-	-	-	tr	tr	tr	+	tr	-	tr	-	+	tr	tr	-	tr	tr	tr
45	Quercetin-trimethyl ether	-	tr	tr	tr	-	-	-	-	tr	-		-	-	-	-	-	-	tr	-
46	Caffeic acid prenyl or isoprenyl ester 1	tr	+	+	tr	+	+++	tr	-	tr	-	+	+	+	tr	-	+	tr	tr	tr
47	Chrysin	+++	+++	+++	+++	+	tr	+	tr	+++	tr	++	++	+	tr	tr	++	+	+++	+
48	Sakuranetin	+	+	+	+	++	+	+	++	tr	-	+	+++	+	+++	++	+	++	+	++
49	Pinocembrin	+++	+++	+++	+++	+	+	+	tr	+	++	++	+	+	tr	tr	+++	++	+++	++
50	Acacetin	-	-	-	-	++	tr	+	++	-	+	+	tr	-	+++	++	-	++	Tr	+
51	Caffeic acid prenyl or isoprenyl derivate 1	tr	-	-	-	tr	-	tr	-	++	-	-	tr	+	tr	tr	-	tr	+	-
52	Pinocembrin chalcone	-	-	-	-	-	-	-	-	+	-	-	+	-	-	-	++	-	-	-
53	Caffeic acid prenyl or isoprenyl ester 2	tr	+	+	+	tr	-	tr	-	tr	-	+	+	tr	tr	-	++	tr	tr	-
54	Caffeic acid benzyl ester	+	+	+	++	tr	++	tr	tr	+	+	+	+	tr	tr	-	-	tr	tr	tr
55	Caffeic acid prenyl or isoprenyl derivate 2	tr	-	-	-	+	+	+	-	-	-	+	++	tr	tr	-	-	tr	+	tr
56	Flavonoid	tr	-	-	-	-	-	tr	+	+	-	-	tr	tr	tr	-	-	-	-	-
57	Genkwanin	-	tr	tr	tr	tr	+	+	+	tr	+	tr	tr	tr	+	+	-	+	tr	+
58	Galangin	+++	+++	+++	+++	++	+	+	tr	++	+	++	++	tr	-	+	+++	+	++	+
59	Kaempheride	tr	tr	tr	tr	++	tr	tr	tr	tr	+	+	tr	tr	tr	++	-	++	tr	++
60	2-Acetyl-1,3-di-*p*-Coumaroylglycerol	tr	tr	tr	tr	++	++	++	+++	+	+++	+	tr	++	+++	+	-	++	+	+
61	3-*O*-acetyl-pinobanksin	+++	++	++	++	+	+	+	-	++	-	++	++	tr	+	-	+++	+	+++	+
62	Quercetin-dimethyl	-	-	-	-	+	-	tr	+	tr	+	tr	-	tr	tr	tr	-	+	-	+
63	* 2-Acetyl-3-*p*-coumaroyl-1-feruloylglycerol	tr	tr	tr	tr	+	+	+	+	tr	+	tr	-	+	+	+	-	+	-	+
64	Metoxychrysin	+	+	+	+	tr	+	tr	-	tr	-	+	tr	tr	tr	-	+	tr	tr	-
**No.**	**Component**	**UK1**	**UK2**	**UK3**	**UT**	**RBH**	**RB1**	**RB2**	**RKH**	**RKK**	**RKP**	**RN**	**RP**	**RS**	**RT**	**RV**	**KR**	**KZ**	**SL**	**PL**
65	* 3-Acetyl-1,2-di-*p*-coumaroylglycerol	tr	-	-		tr	tr	tr	tr	-	tr	tr	-	tr	-	-	-	tr	-	tr
66	Caffeic acid phenethyl ester (CAPE)	+	+	+	+	+	tr	+	-	++	-	+	+	tr	tr	-	-	+	+	-
67	2-Acetyl-1,3-di-feruloylglycerol	tr	-	-	-	tr	tr	tr	+	-	+	tr	-	+	tr	+	-	tr	-	tr
68	Dimethyl luteolin	tr	tr	tr	tr	++	+	+	+	+	++	+	tr	tr	++	++	-	++	tr	+
69	Caffeic acid pentyl or isopentyl ester 1	-	-	-	-	tr	-	tr	-	tr	-	+	+	tr	tr	-	-	-	-	-
70	Flavonoid dimethyl ether	tr	-	-	-	+	-	+	tr	tr	+	-	-	-	+	tr	-	+	-	-
71	*p*-Coumaric acid prenyl ester 1	tr	tr	tr	tr	tr	-	-	-	-	-	-	-	tr	-	-	-	-	tr	
72	*p*-Coumaric acid prenyl ester 2	tr	tr	tr	tr	-	-	-	-	tr	-	tr	-	tr	-	-	-	-	tr	+
73	*p*-Coumaric acid benzyl ester	+	+	+	+	++	+	++	++	+	+	+	-	++	+++	+	-	+	+	++
74	Ferulic or isoferulic acid benzyl ester	+	+	+	+	+	+	+	++	+	+	+	-	+	+	+	-	+	+	+
75	Caffeic acid cinnamic ester	++	tr	tr	tr	tr	tr	tr	-	-	+	tr	-	tr	-	tr	-	tr	+	tr
76	3-*O*-propyl-pinobanksin	+	+	+	tr	-	-	tr	-	+	-	tr	-	tr	-	-	+	tr	+	-
77	*p*-Coumaric acid phenethyl ester	-	-	-	-	-	-	tr	tr	tr	-	-	-	-	-	tr	-	-	tr	+
78	Pinostrobin chalcone	-	tr	tr	tr	tr	-	+	-	+	-	tr	++	tr	-	-	-	tr	tr	+
79	Tectochrysin (Chrysin-7-methyl ether)	+	+	+	+	tr	tr	tr	-	-	-	tr	+	-	-	-	tr	-	+	-
80	Pinostrobin (Pinocembrin-5-methyl ether)	tr	tr	tr	+	+	+++	+	-	+	+	+	++	tr	+	-	tr	+	+	+
81	3-O-butyl or isobutyl pinobanksin	+	+	+	tr	+	tr	-	-	+	-	+	-	-	-	-	-	+	+	tr
82	Galangin-7-methyl ether	+	tr	tr	+	+	+	+	+	tr	+	+	+	-	+	+	-	tr	tr	tr
83	3-*O*-pentyl or isopentyl pinobanksin 1	tr	+	+	tr	+	tr	-	-	tr	-	tr	-	-	-	-	+	tr	tr	-
84	3-*O*-pentyl or isopentyl pinobanksin 2	tr	tr	tr	tr	-	-	-	-	tr	-	tr	-	-	tr	-	-	+	tr	-
85	3-*O*-hexyl- pinobanksin	tr	tr	tr	tr	-	-	-	-	-	-	tr	-	-	-	-	-	tr	tr	-
86	Metoxycinnamic acid cinnamyl ester	tr	tr	tr	tr	+	+	tr	-	tr	-	tr	tr	++	+	-	-	tr	tr	tr

- component absent; + component present in relatively low concentration; ++ component present in relatively average concentration; +++ component present in relatively high concentration; UK1—Ukraine, Khmielnitsky 1; UK2—Ukraine, Khmielnitsky 2; UK3—Ukraine, Khmielnitsky 3; UT—Ukraine, Tarnopol; RB1—Russia, Barnaul 1; RB2—Russia, Barnaul 2; RBH—Russia, Bashikiria; RKP—Russia, Kedrovaja Pad; RKH—Russia, Khabarovsk; RKK—Russia, Krasnodar Krai; RN—Russia, Novosybirsk; RN—Russia, Saratov Oblast; RT—Russia, Tomsk; RV—Russia, Vologoda Oblast; KR—Kyrgyzstan; KZ—Kazahstan, Almastka Oblast; PL—Poland, Lubelszczyzna Region; SN—Slovakia, Nova Bana; * component tentatively identified; ** substitution positioning of glycerol was tentatively identified; ^b^ component identified by comparison with literature; ^c^ component identified by prediction of mass fragment and UV spectrum.

**Table 3:** Colorimetric evaluation of Eurasian propolis.

Country	Plant Origin	TPC [mg GAE/g]	TFC [mg QE/g]	[TPC-TFC] [mg/g]	DPPH [mg GAE/g]	FRAP [mmol Fe^2+^/g]	ORAC [mmol Trx/g]
**UK1**	P	128.43 ± 2.15	55.24 ± 0.82	73.19	36.10 ± 1.40	0.25 ± 0.01	3.85 ± 0.13
**UK2**	P	135.89 ± 1.17	55.79 ± 1.76	80.10	32.83 ± 0.64	0.29 ± 0.02	3.99 ± 0.23
**UK3**	P	120.30 ± 1.22	53.90 ± 2.36	66.40	30.98 ± 0.83	0.34 ± 0.01	4.17 ± 0.16
**UT**	P	145.24 ± 2.24	82.71 ± 2.46	62.53	42.69 ± 0.91	0.40 ± 0.04	4.23 ± 0.24
**RB1**	A-B-P	121.26 ± 0.29	25.84 ± 0.57	95.42	64.47 ± 0.70	1.03 ± 0.02	3.46 ± 0.16
**RB2**	A-B-P	120.02 ± 2.00	26.77±0.71	93.25	53.55 ± 0.31	0.91 ± 0.03	3.56 ± 0.19
**RBH**	A-B-P	48.89 ± 0.37	31.63±2.81	93.25	39.39 ± 0.19	1.28 ± 0.07	3.68 ± 0.27
**RKP**	A-B	50.39 ± 0.43	30.48 ± 0.82	19,91	31.76 ± 2.53	0.13 ± 0.01	3.55 ± 0.22
**RKH**	A-B	52.81 ± 0.41	33.93 ± 1.06	17,26	34.65 ± 2.74	0.15 ± 0.01	3.63 ± 0.17
**RKK**	P	129.21 ± 0.91	53.13 ± 1.26	76.08	29.02 ± 1.60	0.17 ± 0.01	3.56 ± 0.14
**RN**	P	99.98 ± 0.52	41.65 ± 1.58	58.34	25.27 ± 0.73	0.12 ± 0.01	3.67 ± 0.11
**RP**	P	81.65 ± 2.09	27.34 ± 0.94	54.31	32.22 ± 0.39	0.11 ± 0.01	3.96 ± 0.34
**RS**	A-P	109.55 ± 0.49	10.45 ± 0.46	99.10	41.19 ± 1.78	0.08 ± 0.01	4.12 ± 0.26
**RT**	A-B	40.02 ± 0.79	32.49 ± 1.15	7.53	12.59 ± 0.52	0.06 ± 0.01	3.78 ± 0.49
**RV**	A-B-P	47.13 ± 0.50	31.97 ± 0.79	15.16	33.70 ± 0.49	1.17 ± 0.06	4.17 ± 0.12
**KR**	P	30.28 ± 0.46	17.75 ± 0.72	12.52	8.83 ± 0.49	0.10 ± 0.01	1.95 ± 0.36
**KZ**	A-B-P	45.04 ± 0.45	44.89 ± 1.45	0.14	24.67 ± 1.00	0.85 ± 0.03	2.89 ± 0.46
**PL**	A-B-P	46.91 ± 1.10	40.94 ± 1.35	2.05	28.34 ± 0.42	1.15 ± 0.07	3.27 ± 0.38
**SN**	P	42.47 ± 0.81	40.42 ± 1.07	5.97	28.28 ± 0.18	0.92 ± 0.01	3.73 ± 0.15

DPPH—radical scavenging activity in DPPH test; FRAP—ferric reducing antioxidant power; TPC—total phenolic content; mg GAE/g—concentration or activity as mg of gallic acid equivalents per gram of propolis; TFC—flavonoid content; mg QE/g—concentration or activity as mg of quercetin equivalents per gram of propolis; [TPC-TFC]—relatively flavonoids reduced polyphenols content; mmol Fe^2+^/g—activity as mmol of Fe^2+^ equivalents per gram of propolis; UK1—Ukraine, Khmielnitsky 1; UK2—Ukraine, Khmielnitsky 2; UK3—Ukraine, Khmielnitsky 3; UT—Ukraine, Tarnopol; RB1—Russia, Barnaul 1; RB2—Russia, Barnaul 2; RBH—Russia, Bashikiria; RKP—Russia, Kedrovaja Pad; RKH—Russia, Khabarovsk; RKK—Russia, Krasnodar Krai; RN—Russia, Novosybirsk; RN—Russia, Saratov Oblast; RT—Russia, Tomsk; RV—Russia, Vologoda Oblast; KR—Kyrgyzstan; KZ—Kazahstan, Almastka Oblast; PL—Poland, Lubelszczyzna Region; SN—Slovakia, Nova Bana.; P—poplar propolis (trace of non-poplar markers); A-B—aspen–birch propolis (trace of non-aspen markers); A-P—aspen–poplar propolis.

## Data Availability

Not applicable.
